# Increase in Depression and Anxiety Among Australian Gay and Bisexual Men During COVID-19 Restrictions: Findings from a Prospective Online Cohort Study

**DOI:** 10.1007/s10508-021-02276-2

**Published:** 2022-01-17

**Authors:** Benjamin R. Bavinton, Curtis Chan, Mohamed A. Hammoud, Lisa Maher, Bridget Haire, Louisa Degenhardt, Martin Holt, Toby Lea, Nicky Bath, Daniel Storer, Fenyi Jin, Andrew E. Grulich, Adam Bourne, Peter Saxton, Garrett P. Prestage, On behalf of the Flux Research Group, Dean Murphy, Brent Mackie, Colin Batrouney, Jeanne Ellard, Jeffrey Grierson, Marcus Pastorelli

**Affiliations:** 1 Kirby Institute, UNSW Sydney, Level 6, Wallace Wurth Building, Sydney, NSW 2052 Australia; 2 National Drug and Alcohol Research Centre, UNSW Sydney, Sydney, NSW Australia; 3 Centre for Social Research in Health, UNSW Sydney, Sydney, NSW Australia; 4 National LGBTI Health Alliance, Sydney, NSW Australia; 5 Australian Research Centre in Sex, Health and Society, La Trobe University, Melbourne, VIC Australia; 6 School of Population Health, University of Auckland, Auckland, New Zealand

**Keywords:** Gay and bisexual men, Mental health, COVID-19, Depression, Anxiety, Sexual orientation

## Abstract

We examined depression and anxiety prior to and during COVID-19 restrictions in Australian gay and bisexual men (GBM). In an online cohort, a COVID-19-focused survey was conducted in April 2020. During 2019 and in April 2020, 664 GBM completed the Patient Health Questionnaire (PHQ-9, measuring depression) and Generalized Anxiety Disorder Assessment (GAD-7, measuring anxiety). Increased depression and anxiety were defined as a ≥ 5 point increase on the respective scales. Mean PHQ-9 and GAD-7 scores increased between 2019 and 2020 (PHQ-9: from 5.11 in 2019 to 6.55 in 2020; GAD-7: from 3.80 in 2019 to 4.95 in 2020). The proportion of participants with moderate-severe depression (PHQ-9 ≥ 10) increased from 18.8% (*n* = 125) to 25.5% (*n* = 169), while the proportion of participants with moderate-severe anxiety (GAD-7 ≥ 10) increased from 12.7% (*n* = 84) to 17.3% (*n* = 115). Almost one-quarter of participants (*n* = 158, 23.8%) had increased depression; in these men, mean PHQ-9 increased from 2.49 in 2019 to 11.65 in 2020 (*p* < 0.001). One-in-five (20.6%) participants (*n* = 137) had increased anxiety; among these men, mean GAD-7 increased from 2.05 in 2019 to 10.22 in 2020 (*p* < 0.001). Increases were associated with concerns about job security, reduction in social and sexual connections and opportunities, and being personally concerned about COVID-19 itself. COVID-19 appeared to have a sudden and pronounced impact on depression and anxiety in Australian GBM, with a significant minority showing sharp increases. Ongoing monitoring is required to determine longer-term impacts and GBM need access to appropriate and sensitive supports both during and after the COVID-19 pandemic.

**Supplementary Information:**

The online version contains supplementary material available at 10.1007/s10508-021-02276-2.

## Introduction

Severe Acute Respiratory Syndrome coronavirus-2 (SARS-CoV-2), the cause of COVID-19, is highly transmissible through close proximity with an infected person (Guan et al., 2020; Guo et al., 2020). From early March through late April 2020, Australian jurisdictions implemented restrictions in response to COVID-19. During initial “shutdown” periods, people could leave their homes for essential shopping, to provide care or seek medical treatment, to exercise, or to commute to work/study if not possible from home, and were advised to restrict sexual encounters to established intimate partners (COVID-19 National Incident Room Surveillance Team, 2020).

Research in Australia and elsewhere has documented impacts of COVID-19 restrictions on mental health in the general population, including increased feelings of stress and isolation, and producing or exacerbating mental health problems (Fisher et al., 2020; Li et al., 2020; Pierce et al., 2020; Qiu et al., 2020). Social connection and support are protective against declines in mental health during the pandemic (Cao et al., 2020). While the COVID-19 pandemic presents mental health challenges for the general population, physical distancing restrictions may have specific impacts on gay and bisexual men (GBM), who typically have higher prevalence of mental ill-health than the general population (King et al., 2008; Meyer, 2003; Prestage et al., 2018). Restrictions associated with COVID-19 may prevent GBM from accessing critical sexuality-affirming supports (Brennan et al., 2020; Santos et al., 2020; Suen et al., 2020). Peer support among GBM and engagement with gay community have been found to counter negative mental health effects, increase health-seeking behaviors, and assist men to find a sense of belonging (Anderson & Knee, 2021; Hammoud et al., 2019; Mao et al., 2009; Prestage et al., 2018). Cross-sectional research has documented emerging concerns about the mental health of GBM due to COVID-19, with studies during the COVID-19 period finding that just under one-third of GBM experienced depression or anxiety (Santos et al., 2020; Suen et al., 2020), that those who practiced physical distancing were more likely to feel anxious (Holloway et al., 2021), and another reporting that over half felt they needed help with a mental health problem (Brennan et al., 2020). A systematic review from the United Kingdom found that mental health and well-being outcomes were poorer among lesbian, gay, bisexual, and transgender (LGBT) people, but the low data quality was noted (McGowan et al., 2021). Researchers have called for specific monitoring of sexual minorities and mental health during the COVID-19 pandemic (Phillips et al., 2020). In the context of pre-existing, disproportionate burden of mental ill-health among GBM, coupled with a higher likelihood of experiencing co-situated challenges such as stigma and discrimination (Victorian Agency for Health Information, 2020), mental health services (and funders) need to be cognisant of the unique needs of this population during the pandemic.

Our ongoing cohort of Australian GBM provided an opportunity to explore the impact of COVID-19 on depression and anxiety in this population. This analysis aimed to: (1) Determine changes in symptoms of depression and anxiety after the initial implementation of COVID-19 physical distancing restrictions, including trends between 2015 and 2020; and (2) Identify factors associated with increased symptoms of depression and anxiety after the initial implementation of COVID-19 physical distancing restrictions.

## Method

### Participants and Procedure

The *Following Lives Undergoing Change (Flux) Study* is a national, online, prospective observational study of GBM in Australia, launched in 2014. The study protocol has been published (Hammoud et al., 2017). Men were eligible to participate in the study if they were ≥ 16 years of age, identified as gay or bisexual or had sex with a man in the previous 12 months, and lived in Australia. Participants provided informed consent by selecting a checkbox on the online survey indicating they had read and understood the requirements of participating in the study. No compensation was offered for participation. Ethical approval was provided by UNSW Sydney.

The study was promoted by online advertising on social media and gay sexual networking sites/apps. Participants completed online surveys at six-monthly intervals. In early April 2020, participants were invited to complete a survey round with new questions assessing the impact of COVID-19. Men were included in this analysis if they: (1) had participated in a *Flux* survey during 2019 and completed the depression and anxiety measures at least once (for participants with two 2019 surveys, analysis was based on the more recent data); and (2) participated in the 2020 COVID-19 round and provided complete responses to the depression and anxiety measures.

### Measures

Depression was measured using the Patient Health Questionnaire (PHQ-9; scale range = 0–27) (Kroenke et al., 2001) and anxiety was measured using the Generalized Anxiety Disorder Assessment (GAD-7; scale range = 0–21) (Spitzer et al., 2006). Both are well-established instruments and align closely with diagnostic criteria for major depressive disorder and generalized anxiety disorder, respectively (Kroenke et al., 2010). Patients rated each symptom from 0 (not experienced) to 3 (experienced every day). Scale scores were calculated for each participant. A score of 10 on each scale was considered the clinical cut-off for symptoms consistent with depression or anxiety, and were termed “moderate-severe” depression or anxiety, respectively (Kroenke et al., 2001). Following McMillan et al. (2010), a ≥ 5 point increase between 2019 and the 2020 COVID-19 round on the PHQ-9 or GAD-7 was taken as a clinically significant and meaningful increase in symptoms of depression or anxiety, respectively.

Questionnaires included demographic items such as age, country of birth, and sexual identity; self-reported HIV serostatus and testing histories; and sexual behaviors with male partners. A validated measure of sexual sensation-seeking was included to measure the propensity to seek out novel or risky sex (scale range = 11–44) (Kalichman & Rompa, 1995), as was a reliable two-item scale measuring “gay social engagement” (GSE; number of gay friends and amount of time spent with gay men; scale range = 0–7) (Zablotska et al., 2011), Participants reported their postcode of residence. Using a previously published method estimating the proportion of men identifying as gay in every Australian postcode (Callander et al., 2020), we classified a postcode as a “gay suburb” if the proportion of gay-identified men was in the 95th percentile or greater (with an average of approximately 10% of resident men identifying as gay). COVID-19-related items were developed by the study team to measure changes in sexual behaviors, social behaviors, attitudes, employment, and experiences of COVID-19 since physical distancing measures were first implemented in Australia.

### Statistical Analysis

Analysis was conducted using Stata version 14.2 (StataCorp, College Station, TX). Trends among the included men were examined from 2015 to 2020. Mean differences in scale scores were compared using paired-samples *t*-tests; these focused on means from 2019 and 2020, but for sensitivity, also between 2018 and 2020. Factors associated with increased depression and anxiety were identified in bivariate analysis with *t*-tests for continuous variables and chi-square tests for categorical variables. In multivariate analysis, factors independently associated with increased symptoms were identified using logistic regression; we present adjusted odds ratios (aOR), 95% confidence intervals (95% CI) and *p*-values.

## Results

The PHQ-9 and GAD-7 were completed by 913 men in the 2020 COVID-19 survey, and of these, 664 also completed the measures in 2019. Compared to the 664 included men, the 249 men who did not complete them in 2019 were younger (*p* = 0.003); less likely to have a university qualification (*p* = 0.039); and had higher mean scores on the PHQ-9 (*p* = 0.002) and the GAD-7 (*p* = 0.001) in 2020. They were not different in terms of other demographic indicators, HIV status, or mean GSE scale scores. Included participants had a median age of 44 years (IQR = 34–56), and 72.4% (*n* = 481) were university educated. One-quarter (*n* = 172, 25.9%) lived in a “gay postcode.” Most were born in Australia (*n* = 532, 80.1%) and had an Anglo-Celtic ethnicity (*n* = 525, 79.1%). Most were HIV-negative (*n* = 591, 89.0%), while 7.7% (*n* = 51) were HIV-positive, and 3.3% (*n* = 22) were untested or had unknown HIV status. There was a statistically significant decrease in mean GSE scores between 2019 (M = 3.95, SD = 1.54) and 2020 (M = 3.54, SD = 1.53, *p* < 0.001).

### Depression

**Fig. 1:**
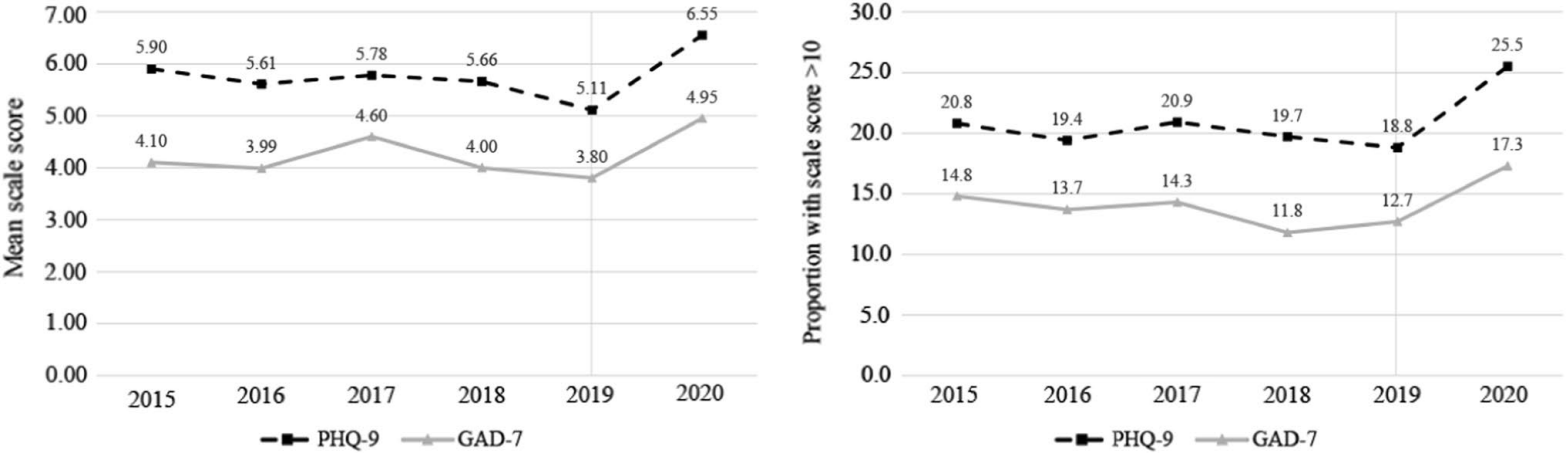
Trends in depression and anxiety, 2015 to 2020: **a** Mean scale score; **b** Proportion of sample with scale score > 10, PHQ-9, Patient Health Questionnaire; GAD-7, Generalized Anxiety Disorder Assessment. The grey line on the figures refers to the final pre-COVID-19 survey round

Figure 1a shows mean PHQ-9 scores over time, from 2015 to 2020, among the 664 men included in the analysis. In the five years before 2020, the average mean PHQ-9 score was 5.61 (range = 5.11–5.90). The mean PHQ-9 score was 5.11 (SD = 5.99) in 2019 and 6.55 (SD = 6.00) in the 2020 COVID-19 survey (*t* = –6.53, *p* < 0.001). To account for the significant decrease between 2018 and 2019 (*t* = 2.69, *p* < 0.001), we also examined the increase between 2018 and 2020, which was also statistically significant (*t* = –3.15, *p* = 0.002). The proportion of participants with moderate-severe depression (i.e. PHQ-9 score of ≥ 10) increased from 18.8% (*n* = 125) to 25.5% (*n* = 169; Fig. 1b). Further details on the scale categories are presented in Supplementary Table S1.

Between 2019 and 2020, 158 participants (23.8%) increased ≥ 5 points on the PHQ-9, indicating reliably increased symptoms of depression (hereafter referred to “increased depression”). Within these 158 men, mean PHQ-9 increased from 2.49 (SD = 4.03) in 2019 to 11.65 (SD = 5.51) in 2020 (*t* = −27.05, *p* < 0.001). While 7.6% (*n* = 12) of these 158 participants had a PHQ-9 of ≥ 10 in 2019, this increased to 55.7% (*n* = 88) in 2020.

In bivariate analyses, compared to the 506 men without increased depression, those with increased depression (*n* = 158) were more likely to: be living with housemates (*p* = 0.028); have been laid off temporarily or permanently from work (*p* = 0.002); be concerned about losing their job due to COVID-19 (*p* < 0.001); have found it very difficult to stop going out for entertainment (*p* = 0.004); have found it very difficult to stop kissing/hugging friends and family (*p* < 0.001); have found it very difficult to not have sex with new partners (*p* = 0.006); have lost contact with people they cared about (*p* < 0.001); be younger (*p* = 0.001); and have had higher mean GSE score in 2019 (*p* = 0.040) (Table 1). They were less likely to have received support from work colleagues (*p* = 0.032) (Table 1). Among the 51 HIV-positive men, very few (*n* = 6) reported difficulties accessing their HIV medications, and this was not associated with increased depression (*p* = 0.502). Similarly, in total only two HIV-positive participants experienced changes in their viral load, and this was also not associated with increased depression (*p* = 0.781). In multivariate logistic regression, factors independently associated with increased depression were: concern about losing their job (aOR = 2.03, 95% CI = 1.19–3.48, *p* = 0.009); having lost contact with people they cared about (aOR = 2.28, 95% CI = 1.32–3.92, *p* = 0.003); and a higher GSE in 2019 (aOR = 1.30, 95% CI = 1.10–1.54, *p* = 0.003) (Table 2).

**Table 1:** Bivariate associations with increased symptoms consistent with depression (≥ 5-point increase in PHQ-9^a^ score) and increased symptoms consistent with anxiety (≥ 5-point increase in GAD-7^b^ score)

	Symptoms of depression			Symptoms of anxiety		
	Did not increase (*n* = 506)	Increased (*n* = 158)	*p*-value	Did not increase (n = 527)	Increased (n = 137)	*p*-value
*N (%)*						
Employment prior to COVID-19			0.200			0.139
Full-time	331 (65.4)	99 (62.7)		345 (65.5)	85 (62.0)	
Part-time or casual	90 (17.8)	36 (22.8)		93 (17.7)	33 (24.1)	
Studying	13 (2.8)	7 (4.4)		14 (2.7)	6 (4.4)	
Not working	72 (14.2)	16 (10.1)		75 (14.2)	13 (9.5)	
University education	370 (73.1)	111 (70.3)	0.481	384 (72.9)	97 (70.8)	0.630
Lives in “gay postcode”	128 (25.3)	44 (27.9)	0.523	131 (24.9)	41 (29.9)	0.228
Born in Australia	410 (81.0)	122 (77.2)	0.295	427 (81.0)	105 (76.6)	0.252
HIV status			0.286			0.357
HIV-positive	43 (8.5)	8 (5.1)		39 (7.4)	12 (8.8)	
HIV-negative	445 (87.9)	146 (92.4)		468 (88.8)	123 (89.8)	
Untested/unknown HIV status	18 (3.56)	4 (2.5)		20 (3.8)	2 (1.5)	
Relationship status and living situation			0.681			0.101
No boyfriend/husband	236 (46.6)	77 (48.7)		257 (48.8)	56 (40.9)	
Had boyfriend/husband, living separately	75 (14.8)	26 (16.5)		73 (13.9)	28 (20.4)	
Had boyfriend/husband, living together	195 (38.5)	55 (34.8)		197 (37.4)	53 (38.7)	
Living with						
Boyfriend/husband	195 (38.5)	55 (34.8)	0.399	197 (37.4)	53 (38.7)	0.779
Gay friends	35 (6.9)	15 (9.5)	0.284	37 (7.0)	13 (9.5)	0.329
Other friends	20 (4.0)	8 (5.1)	0.544	22 (4.2)	6 (4.4)	0.915
Family	73 (14.4)	17 (10.5)	0.240	77 (14.6)	13 (9.5)	0.119
Housemates	**56 (11.1)**	**28 (17.7)**	**0.028**	62 (11.8)	22 (16.1)	0.178
No one else	132 (26.1)	45 (28.5)	0.552	139 (26.4)	38 (27.7)	0.748
Less sex with casual partners since COVID-19	333 (65.8)	101 (63.9)	0.664	348 (66.0)	86 (62.8)	0.475
Less sex with fuckbuddies since COVID-19	363 (71.7)	120 (76.0)	0.300	381 (72.3)	102 (74.5)	0.614
Laid off from work temporarily or permanently	**52 (10.3)**	**31 (19.6)**	**0.002**	65 (12.3)	18 (13.1)	0.800
Concerned about losing job due to COVID-19	**93 (18.4)**	**51 (32.3)**	** < 0.001**	**102 (19.4)**	**42 (30.7)**	**0.004**
No social activities in the last week	364 (72.1)	106 (67.1)	0.262	368 (70.0)	102 (74.5)	0.515
Finding it very difficult to:						
Stop going out for entertainment	**138 (27.3)**	**62 (39.2)**	**0.004**	151 (28.7)	49 (35.8)	0.106
Stop kissing/hugging friends and family	**181 (35.8)**	**82 (51.9)**	** < 0.001**	**190 (36.1)**	**73 (53.3)**	** < 0.001**
Not have sex with new partners	**90 (17.8)**	**44 (27.9)**	**0.006**	**97 (18.4)**	**37 (27.0)**	**0.025**
Concerned about						
Contracting COVID-19	241 (47.6)	81 (51.3)	0.425	**240 (45.5)**	**82 (59.9)**	**0.003**
Transmitting COVID-19 to others	362 (71.5)	123 (77.9)	0.119	**373 (70.8)**	**112 (81.8)**	**0.010**
Getting sick with COVID-19	258 (51.0)	88 (55.7)	0.301	**262 (49.7)**	**84 (61.3)**	**0.015**
Overwhelming the health system	431 (85.2)	139 (88.0)	0.379	**445 (84.4)**	**125 (91.2)**	**0.042**
Agreement with: “I have lost contact with people I care about”	**209 (41.3)**	**102 (64.6)**	** < 0.001**	**229 (43.5)**	**82 (59.9)**	**0.001**
*Mean (SD*^*c*^*)*						
Age	**45.7 (13.6)**	**41.6 (12.2)**	**0.001**	45.1 (13.7)	43.3 (12.2)	0.157
Sexual sensation-seeking scale (2019)	30.5 (6.1)	31.4 (6.1)	0.173	**30.4 (6.0)**	**32.0 (6.4)**	**0.030**
Gay social engagement scale (2019)	**3.9 (1.5)**	**4.2 (1.5)**	**0.040**	3.9 (1.5)	4.2 (1.6)	0.153
Gay social engagement scale (2020)	3.5 (1.5)	3.5 (1.6)	0.953	3.5 (1.5)	3.5 (1.6)	0.764
Level of support during COVID-19 received from						
Family	2.16 (0.87)	2.03 (0.89)	0.113	2.03 (0.98)	1.92 (0.99)	0.248
Gay friends	2.00 (0.85)	1.97 (0.85)	0.697	1.87 (0.97)	1.76 (0.97)	0.221
Straight friends	2.05 (0.80)	1.90 (0.85)	0.053	1.89 (0.94)	1.72 (0.98)	0.070
Boyfriend/husband	2.56 (0.82)	2.45 (0.83)	0.256	1.36 (1.41)	1.46 (1.38)	0.469
Sex partners	1.24 (1.09)	1.12 (1.03)	0.400	0.59 (0.96)	0.45 (0.87)	0.115
Work colleagues	**2.14 (0.89)**	**1.95 (0.93)**	**0.032**	1.74 (1.16)	1.69 (1.08)	0.610
Neighbors	1.00 (1.05)	0.82 (0.96)	0.080	0.79 (1.02)	0.75 (0.97)	0.727
People on social media	1.55 (1.03)	1.47 (0.92)	0.418	1.32 (1.09)	1.34 (0.98)	0.856
Housemates	1.84 (1.21)	1.88 (1.13)	0.841	0.70 (1.15)	0.72 (1.19)	0.814

Significant values (*p* < 0.05) are given in bold

^a^PHQ-9, Patient Health Questionnaire

^b^GAD-7, Generalized Anxiety Disorder Assessment

^c^SD, standard deviation

**Table 2:** Multivariate associations with increased symptoms consistent with depression (≥ 5-point increase in PHQ-9^a^ score) and increased symptoms consistent with anxiety (≥ 5-point increase in GAD-7^b^ score)

	Symptoms of depression			Symptoms of anxiety		
	aOR^c^	95% CI^d^	*p*-value	aOR^c^	95% CI^d^	*p*-value
*N (%)*						
Living with housemates	2.11	0.95–3.25	0.075	–	–	–
Laid off temporarily or permanently	1.46	0.71–2.99	0.299	–	–	–
Concerned about losing job due to COVID-19	**2.03**	**1.19–3.48**	**0.009**	1.26	0.72–2.23	0.419
Finding it very difficult to						
Stop going out for entertainment	1.53	0.88–2.70	0.133	–	–	–
Stop kissing/hugging friends and family	1.49	0.87–2.57	0.150	**1.83**	**1.09–3.09**	**0.022**
Not have sex with new partners	1.04	0.56–1.95	0.894	1.06	0.58–1.94	0.845
Concerned about						
Contracting COVID-19	–	–	–	1.45	0.70–3.00	0.320
Transmitting COVID-19 to others	–	–	–	0.95	0.49–1.83	0.882
Getting sick with COVID-19	–	–	–	0.95	0.46–1.96	0.883
Overwhelming the health system	–	–	–	**2.98**	**1.02–8.76**	**0.047**
Agreement with: “I have lost contact with people I care about”	**2.28**	**1.32–3.92**	**0.003**	1.16	0.64–2.13	0.621
*Mean (SD*^*e*^*)*						
Age	0.98	0.96–1.00	0.129	–	–	–
Sexual sensation-seeking scale	–	–	–	1.04	0.99–1.08	0.106
Gay social engagement (2019)	**1.30**	**1.10–1.54**	**0.003**	–	–	–
Level of support received from work colleagues	0.78	0.59–1.03	0.080	–	–	–

Significant values (*p* < 0.05) are given in bold

^a^PHQ-9, Patient Health Questionnaire

^b^GAD-7, Generalized Anxiety Disorder Assessment

^c^aOR, adjusted odds ratio

^d^CI, confidence interval

^e^SD, standard deviation

### Anxiety

Between 2015 and 2019, mean GAD-7 scores averaged at 4.10 (range = 3.80–4.60; Fig. 1a). Mean GAD-7 was 3.80 (SD = 4.78) in 2019 and increased to 4.95 (SD = 5.05) in 2020 (*t* = –6.05, *p* < 0.001). Again, to account for the drop between 2018 and 2019 (*t* = 1.12, *p* < 0.001), the increase between 2018 and 2020 was also statistically significant (*t* = –3.81, *p* < 0.001). The proportion of participants with moderate-severe anxiety (i.e. GAD-7 score of ≥ 10) increased from 12.7% (*n* = 84) to 17.3% (*n* = 115; Fig. 1b). Further details on the scale categories are presented in Supplementary Table S1.

One-in-five (*n* = 137) participants increased ≥ 5 points on the GAD-7, indicating reliably increased symptoms of anxiety (hereafter referred to as ‘increased anxiety’). Within these 137 men, mean GAD-7 increased from 2.05 (SD = 3.48) in 2019 to 10.22 (SD = 4.69) in 2020 (*t* = −27.27, *p* < 0.001). While only 5.8% (*n* = 8) of these 137 participants had a GAD-7 of ≥ 10 in 2019, this increased to 41.6% (*n* = 58) in 2020.

In bivariate analyses, compared to those without increased anxiety (*n* = 527), those with increased anxiety (*n* = 137): were concerned about losing their job due to COVID-19 (*p* = 0.004); found it very difficult to stop kissing/hugging friends and family (*p* < 0.001); found it very difficult to not have sex with new partners (*p* = 0.025); were concerned about contracting COVID-19 (*p* = 0.003); were concerned about transmitting COVID-19 to others (*p* = 0.010); were concerned about getting sick with COVID-19 (*p* = 0.015); were concerned about overwhelming the health system (*p* = 0.042); had lost contact with people they cared about (*p* < 0.001); and scored higher on sexual sensation-seeking in 2019 (*p* = 0.030) (Table 1). Among the 51 HIV-positive men, difficulty accessing HIV medication (*p* = 0.273) and change in viral load (*p* = 0.496) were not associated with increased anxiety. In multivariate logistic regression, factors independently associated with increased symptoms of anxiety were: finding it very difficult to stop hugging/kissing friends and family (aOR = 1.83, 95% CI = 1.09–3.09, *p* = 0.022); and concern about overwhelming the health system (aOR = 2.98, 95% CI = 1.02–8.76, *p* = 0.047) (Table 2).

## Discussion

Our results showed an increase in both depression and anxiety among GBM, coinciding with the COVID-19 pandemic and introduction of physical distancing restrictions in Australia. To our knowledge, these are the first such longitudinal data among GBM internationally. We found pre-COVID-19 levels of depression and anxiety were higher in our sample of GBM than in general population studies using the same measures (Stocker et al., 2021). General population studies in high-income countries have reported the prevalence of moderate-to-severe depression at 3.0%–10.8% and moderate-to-severe anxiety at 6.9%–10.6% (Stocker et al., 2021). By contrast, studies using the same measures in GBM have reported prevalence of depression at 12.5%–28.3% (Ferlatte et al., 2019; Prestage et al., 2018; Sewell et al., 2017) and of anxiety at 10.6%–18.0% (Prestage et al., 2018; Sewell et al., 2017). In Australia, a 2007 national survey found that homosexual/bisexual participants had 3.2 times the prevalence of affective disorders (including depression) and 2.2 times the prevalence of anxiety disorders compared to heterosexual participants (Australian Bureau of Statistics, 2008).

From these already high baseline levels, we observed a 28% relative increase in PHQ-9 mean scores and a 30% relative increase in GAD-7 mean scores between 2019 and the 2020 COVID-19 survey. In 2020, one-quarter of participants screened positively for moderate-severe depression and 17% moderate-severe anxiety, similar to levels found in cross-sectional studies of the general Australian population during COVID-19 (Dawel et al., 2020; Fisher et al., 2020). Nearly one-quarter of participants had increased depression, while one-fifth had increased anxiety. These changes were associated with a range of factors including concerns about job security and work, reduction in social and sexual connection or opportunities, and concerns related to acquiring or transmitting COVID-19 or impacts on the health system.

Some studies examined mental health among GBM in the early COVID-19 pandemic. In an online survey of lesbian, gay, and bisexual people in Hong Kong, 31.5% of participants met the condition for clinical depression and 27.9% for anxiety, while 34.7% reported reduced contact and connection with other GBM due to COVID-19 restrictions (Suen et al., 2020). A global online survey of 2732 GBM across 103 countries conducted in April–May 2020 found that 35% screened positive for depression and 34% for anxiety (Santos et al., 2020). These studies reported higher prevalence than found in our data, but the participants were sampled from different countries with varying COVID-19 epidemics and human rights frameworks. As cross-sectional studies like these could not measure depression and anxiety pre- and post-COVID-19 restrictions, it limits their ability to attribute mental health problems to COVID-19 or other factors (Suen et al., 2020). In a large population-based longitudinal study of the general population in the United Kingdom (UK), clinically significant levels of mental distress rose from 18.9% in 2018–19 to 27.3% one month into the COVID-19 restrictions in the UK; it was reported that the increase in mean scores on the General Health Questionnaire (GHQ-12) was greater than expected by the pre-existing trend (Pierce et al., 2020). Outside of our study, similar longitudinal data do not appear to be available for GBM.

Several factors relating to employment were associated with increased depression/anxiety. Losing employment due to COVID-19 was associated with depression, while concern about losing employment was associated with both depression and anxiety (including at the multivariate level for depression). This supports other research in the general population (Fisher et al., 2020; Pierce et al., 2020) and with sexual minorities (Suen et al., 2020), showing that employment-related impacts are an important mental health stressor during the pandemic.

Concerns about COVID-19 itself, such as being worried about contracting it, transmitting it to others, getting sick, or overwhelming the health system, were associated with increased anxiety but not depression. In multivariate analysis, concern about overwhelming the health system was one of only two factors independently associated with increased anxiety. In both those with increased symptoms of anxiety and those without, the factors relating to concern for others (i.e. transmitting to others and overwhelming the health system) were of greater concern to participants than factors relating to concern for the self (i.e. contracting and getting sick with COVID-19).

Interestingly, while some factors related to sexual practice were associated with increased depression/anxiety, such as finding it very difficult to forego sex with new partners (associated with both depression and anxiety) or sexual sensation-seeking (associated with anxiety), actual reductions in sex with casual partners or “fuckbuddies” during COVID-19 restrictions were not. The mental health effects of COVID-19 restrictions thus appeared to be more related to desire and expectations about sex rather than actual changes in behavior. Relationship status was also not associated with increased depression or anxiety, counter to some international research showing that being in a relationship is protective against depression (Suen et al., 2020). On the other hand, greater social engagement with other gay men prior to (but not during) COVID-19 was strongly associated with increased depressive symptoms, suggesting that men who socialized more with gay friends before COVID-19 struggled more with the restrictions than men who were less socially engaged. Alternatively, it could be that protective aspects of spending time with other gay men (such as camaraderie, enjoyment, and support) were disrupted. Items relating to loneliness more generally were associated with increased symptoms of both depression and anxiety (Dawel et al., 2020), as was experiencing difficulties due to reducing physical contact (hugging/kissing) with friends and family.

Initial increases in depression and anxiety among GBM after physical distancing restrictions were introduced may represent a short-term “spike,” which may fall as people adjust or restrictions ease (Pierce et al., 2020). However, other trends may also occur, such as stabilization at a higher level, or even further increases (e.g. if restrictions are reimposed, if restrictions continue for extended periods, or if there are delays in vaccine distribution). Differential geographic impacts of COVID-19 are highly likely to be relevant. In Australia, physical distancing restrictions are largely implemented at the state level. While all jurisdictions had initial success in containing COVID-19, they had varying levels of success thereafter. More populous states such as New South Wales and Victoria experienced stricter restrictions. In particular, residents of Melbourne, Victoria, were placed under one of the strictest lockdowns globally from July to October 2020 (Victoria State Government, 2020), impacting the ability to make and maintain social and sexual connections and placing limitations on employment for many people. We will examine these issues in a future analysis of an ongoing COVID-19-related extension to the *Flux Study* whereby participants were invited to complete the PHQ-9 and GAD-7 every four weeks (Prestage et al., 2020).

Some implications arose from our analysis. Generally, the factors associated with increased anxiety and depression in our sample of GBM were ones that likely apply to all populations rather than being specific to GBM, such as those related to employment or loss of social contact. However, although it appeared from our data that COVID-19 did not detrimentally affect GBM to a greater extent than the general population, it is important to acknowledge that experiences of sexuality-related stigma and discrimination may impact GBM’s access to appropriate mental health services. Previous research has indicated that GBM have struggled to access sexuality-affirming and culturally safe mental health support (Waling et al., 2019). COVID-19 has highlighted why there is a critical need for generalist mental health support services to be culturally sensitive to diverse populations and where possible, indicate to prospective clients that their services are GBM-friendly. Such services should consider telehealth approaches to avoid disruptions to mental health care. Our finding that higher gay male-specific social engagement prior to COVID-19 was associated with increased depression has suggested that there may be an important role for lesbian, gay, bisexual, and transgender (LGBT) community organizations during periods of COVID-19 restrictions in providing online social support and community connection.

While a significant strength of our analysis was the capacity to compare the mental health of GBM during the first month of COVID-19 restrictions to previous mental health data in the same cohort, the analysis has some limitations. We used an online convenience sample and findings may not be representative of all GBM men in Australia; the sample was somewhat older than other typical samples of Australian GBM (Bavinton et al., 2020), was highly educated, and comprised mostly Australian-born men. The COVID-19 survey instrument itself may not have included all pertinent variables related to mental health among GBM, including quality of relationships and experiences of homophobia, given that the *Flux Study* is primarily focused on HIV prevention, sexual practices, and drug use. Measures related to experiences of COVID-19 were not validated, but due to the rapid onset of COVID-19 and the desire to implement rapid monitoring, this was unavoidable and likely affects most early COVID-19 research studies. Although a longitudinal study, we cannot be certain that COVID-19 caused changes in depression and anxiety, especially given that the pandemic occurred immediately after some months of bushfires that could also have impacted mental health for some Australians.

### Conclusion

COVID-19, and the associated introduction of physical distancing restrictions in Australia, had a sudden and pronounced impact on the mental health of GBM, with increases in both depression and anxiety observed. Ongoing monitoring is required to determine the longer-term impacts, including to examine protective factors associated with resilience. While most factors associated with increased depression and anxiety were relatively general and not specific to GBM, given high baseline prevalence of mental health issues in this population, it is critical to ensure GBM have access to appropriate and sensitive supports as well as evidence-based interventions both during and after the COVID-19 pandemic.

## Supplementary Information

Below is the link to the electronic supplementary material.

Supplementary file1 (DOCX 20 kb)

## Data Availability

Not applicable.

## References

[CR1] Anderson AR, Knee E (2021). Queer isolation or queering isolation? Reflecting upon the ramifications of COVID-19 on the future of queer leisure spaces. Leisure Sciences.

[CR2] Australian Bureau of Statistics (2008). National survey of mental health and wellbeing: Summary of results.

[CR3] Bavinton BR, Grulich AE, Broady T, Keen P, Mao L, Patel P, Chan C, Prestage GP, Holt M (2020). Increases in HIV testing frequency in Australian gay and bisexual men are concentrated among PrEP users: An analysis of Australian behavioural surveillance data, 2013–2018. AIDS and Behavior.

[CR4] Brennan DJ, Card KG, Collict D, Jollimore J, Lachowsky NJ (2020). How might social distancing impact gay, bisexual, queer, trans and Two-Spirit men in Canada?. AIDS and Behavior.

[CR5] Callander D, Mooney-Somers J, Keen P, Guy R, Duck T, Bavinton BR, Grulich AE, Holt M, Prestage GP (2020). Australian 'gayborhoods' and 'lesborhoods': A new method for estimating the number of adult gay men and lesbian women living in each Australian postcode. International Journal of Geographical Information Science.

[CR6] Cao W, Fang Z, Hou G, Han M, Xu X, Dong J, Zheng J (2020). The psychological impact of the COVID-19 epidemic on college students in China. Psychiatry Research.

[CR7] COVID-19 National Incident Room Surveillance Team. (2020). COVID-19, Australia: Epidemiology Report 14 (Reporting week ending 23:59 AEST 3 May 2020). *Communicable Diseases Intelligence,**44*. 10.33321/cdi.2020.44.42 10.33321/cdi.2020.44.42 32393161

[CR8] Dawel A, Shou Y, Smithson M, Cherbuin N, Banfield M, Calear AL, Farrer LM, Gray D, Gulliver A, Housen T, McCallum SM (2020). The effect of COVID-19 on mental health and wellbeing in a representative sample of Australian adults. Frontiers in Psychiatry.

[CR9] Ferlatte O, Salway T, Rice SM, Oliffe JL, Knight R, Ogrodniczuk JS (2019). Inequities in depression within a population of sexual and gender minorities. Journal of Mental Health.

[CR10] Fisher JR, Tran TD, Hammarberg K, Sastry J, Nguyen H, Rowe H, Popplestone S, Stocker R, Stubber C, Kirkman M (2020). Mental health of people in Australia in the first month of COVID-19 restrictions: A national survey. Medical Journal of Australia.

[CR11] Guan WJ, Ni ZY, Hu Y, Liang WH, Ou CQ, He JX, Liu L, Shan H, Lei CL, Hui DS, Du B (2020). Clinical characteristics of coronavirus disease 2019 in China. New England Journal of Medicine.

[CR12] Guo YR, Cao QD, Hong ZS, Tan YY, Chen SD, Jin HJ, Tan KS, Wang DY, Yan Y (2020). The origin, transmission and clinical therapies on coronavirus disease 2019 (COVID-19) outbreak–an update on the status. Military Medical Research.

[CR13] Hammoud MA, Jin F, Degenhardt L, Lea T, Maher L, Grierson J, Mackie B, Pastorelli M, Batrouney C, Bath N, Bradley J (2017). Following Lives Undergoing Change (Flux) Study: Implementation and baseline prevalence of drug use in an online cohort study of gay and bisexual men in Australia. International Journal of Drug Policy.

[CR14] Hammoud MA, Vaccher S, Jin F, Bourne A, Maher L, Holt M, Bavinton BR, Haire B, Degenhardt L, Grulich A, Prestage GP (2019). HIV pre-exposure prophylaxis (PrEP) uptake among gay and bisexual men in Australia and factors associated with the nonuse of PrEP among eligible men: Results from a prospective cohort study. Journal of Acquired Immune Deficiency Syndromes.

[CR15] Holloway IW, Garner A, Tan D, Ochoa AM, Santos GM, Howell S (2021). Associations between physical distancing and mental health, sexual health and technology use among gay, bisexual and other men who have sex with men during the COVID-19 pandemic. Journal of Homosexuality.

[CR16] Kalichman SC, Rompa D (1995). Sexual sensation seeking and sexual compulsivity scales: Validity, and predicting HIV risk behavior. Journal of Personality Assessment.

[CR17] King M, Semlyen J, Tai SS, Killaspy H, Osborn D, Popelyuk D, Nazareth I (2008). A systematic review of mental disorder, suicide, and deliberate self harm in lesbian, gay and bisexual people. BMC Psychiatry.

[CR18] Kroenke K, Spitzer RL, Williams JB (2001). The PHQ-9: Validity of a brief depression severity measure. Journal of General Internal Medicine.

[CR19] Kroenke K, Spitzer RL, Williams JB, Löwe B (2010). The Patient Health Questionnaire Somatic, Anxiety, and Depressive Symptom Scales: A systematic review. General Hospital Psychiatry.

[CR20] Li S, Wang Y, Xue J, Zhao N, Zhu T (2020). The impact of COVID-19 epidemic declaration on psychological consequences: A study on active Weibo users. International Journal of Environmental Research and Public Health.

[CR21] Mao L, Kidd MR, Rogers G, Andrews G, Newman CE, Booth A, Saltman DC, Kippax SC (2009). Social factors associated with major depressive disorder in homosexually active, gay men attending general practices in urban Australia. Australian and New Zealand Journal of Public Health.

[CR22] McGowan VJ, Lowther HJ, Meads C (2021). Life under COVID-19 for LGBT+ people in the UK: Systematic review of UK research on the impact of COVID-19 on sexual and gender minority populations. BMJ Open.

[CR23] McMillan D, Gilbody S, Richards D (2010). Defining successful treatment outcome in depression using the PHQ-9: A comparison of methods. Journal of Affective Disorders.

[CR24] Meyer IH (2003). Prejudice, social stress, and mental health in lesbian, gay, and bisexual populations: Conceptual issues and research evidence. Psychological Bulletin.

[CR25] Phillips Ii G, Felt D, Ruprecht MM, Wang X, Xu J, Pérez-Bill E, Bagnarol RM, Roth J, Curry CW, Beach LB (2020). Addressing the disproportionate impacts of the COVID-19 pandemic on sexual and gender minority populations in the united states: Actions toward equity. LGBT Health.

[CR26] Pierce M, Hope H, Ford T, Hatch S, Hotopf M, John A, Kontopantelis E, Webb R, Wessely S, McManus S, Abel KM (2020). Mental health before and during the COVID-19 pandemic: A longitudinal probability sample survey of the UK population. The Lancet Psychiatry.

[CR27] Prestage GP, Hammoud MA, Jin F, Degenhardt L, Bourne A, Maher L (2018). Mental health, drug use and sexual risk behavior among gay and bisexual men. International Journal of Drug Policy.

[CR28] Prestage, G. P., Storer, D., Murphy, D., Maher, L., Haire, B., Jin, F., Bavinton, B. R., Holt, M., Ellard, J., Grulich, A. E., & Hammoud, M. A. (2020). *Week-on-week changes in sexual behaviour and PrEP use among Australian gay and bisexual men during COVID-19 restrictions*. Paper presented at the Joint Australasian HIV/AIDS and Sexual Health Conference: Virtual, Australia.

[CR29] Qiu J, Shen B, Zhao M, Wang Z, Xie B, Xu Y (2020). A nationwide survey of psychological distress among Chinese people in the COVID-19 epidemic: Implications and policy recommendations. General Psychiatry.

[CR30] Santos, G., Ackerman, B., Rao, A., Wallach, S., Ayala, G., Lamontage, E., Garner, A., Holloway, I. W., Arreola, S., Silenzio, V., Strömdahl, S., Yu. L, Strong, C., Adamson, T., Yakusik, A., Doan, T. T., Huang, P., Cerasuolo, D., Bishop, A., Noori, T., Pharris, A., Aung, M., Dara, M., Chung, S. Y., Hanley, M., Baral, S., Beyrer, C., & Howell, S. (2020). Economic, mental health, HIV prevention and HIV treatment Impacts of COVID-19 and the COVID-19 response on a global sample of cisgender gay men and other men who have sex with men. *Research Square Preprint*. 10.1007/s10461-020-02969-0 PMC7352092 32654021

[CR31] Sewell J, Miltz A, Lampe FC, Cambiano V, Speakman A, Phillips AN, Stuart D, Gilson R, Asboe D, Nwokolo N, Clarke A (2017). Poly drug use, chemsex drug use, and associations with sexual risk behaviour in HIV-negative men who have sex with men attending sexual health clinics. International Journal of Drug Policy.

[CR32] Spitzer RL, Kroenke K, Williams JB, Löwe B (2006). A brief measure for assessing generalized anxiety disorder: The GAD-7. Archives of Internal Medicine.

[CR33] Stocker R, Tran T, Hammarberg K, Nguyen H, Rowe H, Fisher J (2021). Patient Health Questionnaire 9 (PHQ-9) and General Anxiety Disorder 7 (GAD-7) data contributed by 13,829 respondents to a national survey about COVID-19 restrictions in Australia. Psychiatry Research.

[CR34] Suen YT, Chan RC, Wong EMY (2020). Effects of general and sexual minority-specific COVID-19-related stressors on the mental health of lesbian, gay, and bisexual people in Hong Kong. Psychiatry Research.

[CR35] Victoria State Government. (2020). *Statement from the Premier* [Press release]. Retrieved from https://www.premier.vic.gov.au/statement-premier-74.

[CR36] Victorian Agency for Health Information (2020). The health and wellbeing of the lesbian, gay, bisexual, transgender, intersex and queer population in Victoria: Findings from the Victorian Population Health Survey 2017.

[CR37] Waling, A., Lim, G., Dhalla, S., Lyons, A., & Bourne, A. (2019). *Understanding LGBTI+ lives in crisis*. Bundoora, Victoria & Canberra, ACT: Australian Research Centre in Sex, Health and Society and Lifeline Research Foundation.

[CR38] Zablotska IB, Kippax S, Grulich A, Holt M, Prestage G (2011). Behavioural surveillance among gay men in Australia: Methods, findings and policy implications for the prevention of HIV and other sexually transmissible infections. Sexual Health.

