# Supplementary material for: Increase in Depression and Anxiety Among Australian Gay and Bisexual Men During COVID-19 Restrictions: Findings from a Prospective Online Cohort Study
Source: Arch Sex Behav. 2022 Jan 17;51(1):355–64. doi: 10.1007/s10508-021-02276-2 (PMC8763302; doi:10.1007/s10508-021-02276-2)
Supplement: Supplementary file 1 — Supplementary file1 (DOCX 20 kb) [file 10508_2021_2276_MOESM1_ESM.docx]

**Supplementary Table S1. Categories of depression (PHQ-9) and anxiety (GAD-7) among (a) all men in the sample and (b) men with a ≥5-point increase on the respective scale.**

| **N (%)** | **All men in the sample (n=664)** | | **Men with a ≥5-point increase on the respective scale (n=158 for depression; n=137 for anxiety)** | |
| --- | --- | --- | --- | --- |
|  | **2019** | **2020** | **2019** | **2020** |
| **Depression categories (PHQ-9 scores)** |  |  |  |  |
| No/minimal depression (0–4) | 389 (58.6) | 297 (44.7) | 125 (79.1) | 0 (0.0) |
| Mild depression (5–9) | 150 (22.6) | 198 (29.8) | 21 (13.3) | 70 (44.3) |
| Moderate depression (10–14) | 63 (9.5) | 88 (13.3) | 10 (6.3) | 40 (25.3) |
| Moderately-severe depression (15–19) | 37 (5.6) | 53 (8.0) | 1 (0.6) | 32 (20.3) |
| Severe depression (20–27) | 25 (3.8) | 28 (4.2) | 1 (0.6) | 16 (10.1) |
| **Anxiety categories (GAD-7 scores)** |  |  |  |  |
| No/mild anxiety (0–4) | 439 (66.1) | 374 (56.3) | 111 (62.2) | 0 (0.0) |
| Mild anxiety (5–9) | 141 (21.2) | 198 (26.4) | 123 (23.3) | 80 (58.4) |
| Moderate anxiety (10–14) | 53 (8.0) | 75 (11.3) | 46 (8.7) | 31 (22.6) |
| Severe anxiety (15–21) | 31 (4.7) | 40 (6.0) | 30 (5.7) | 26 (19.0) |
